# Transcranial direct current stimulation in management of pain, mood, functionality, and quality of life in patients undergoing hemodialysis: a study protocol for a double-blind controlled randomized trial

**DOI:** 10.1186/s13063-019-3769-6

**Published:** 2019-12-30

**Authors:** Artur Quintiliano, Tayanne Oehmen, Gianna Mastroianni Kirsztajn, Rodrigo Pegado

**Affiliations:** 1 Department of Medicine, Federal University of Rio Grande do Norte, Rio Grande do Norte, 620 Nilo Peçanha St., Natal, RN 59012-300 Brazil; 2 Department of Medicine (Nephrology), Federal University of Sao Paulo, São Paulo, Brazil; 3 Instituto do Rim, Natal, Brazil; 4 Graduate Program in Rehabilitation Science, Federal University of Rio Grande do Norte, Natal, Rio Grande do Norte Brazil

**Keywords:** Brain stimulation, Kidney diseases, Pain, Depression, Quality of life

## Abstract

**Background:**

Persistent pain can lead to incapacitation requiring long-term pharmacological treatment. Up to 82% of chronic kidney disease (CKD) patients undergoing hemodialysis (HD) have chronic pain and most do not respond to usual medication. Advances in non-pharmacological treatments are necessary to promote pain relief without side effects and to restore functionality. Transcranial direct current stimulation (tDCS) promises to be a novel, cost-efficient, non-pharmacological treatment for CKD patients with chronic pain. In this study, we hypothesize that tDCS could improve pain, depression, functionality, and quality of life in patients with CKD undergoing HD.

**Methods/design:**

We describe a single-center, parallel-design, double blind randomized, sham-controlled trial. Forty-five subjects with CKD undergoing HD will be randomized to a motor cortex (M1), a dorso lateral prefrontal cortex (DLPFC), or a sham group. A total of ten sessions will be administered to participants over 4 weeks using a monophasic continuous current with an intensity of 2 mA for 20 min. Participants will be evaluated at baseline, immediately after the tenth session, and at 1 week and 4 weeks of follow-up after the intervention. Pain, depression, functionality, and quality of life will be evaluated.

**Discussion:**

The results from this study will provide initial clinical evidence on the efficacy and safety of tDCS in patients with CKD undergoing HD.

**Trial registration:**

Brazilian Clinical Trials Registry/Registro Brasileiro de Ensaios Clínicos (ensaiosclinicos.gov.br), 1111–1216-0137. Registered on 20 June 2018.

## Background

A complete physical and mood assessment is critical to provide an appropriate treatment plan for chronic kidney disease (CKD) [1]. Management of persistent pain in this population requires an adequate understanding of the pathogenesis and clinical conditions with the aim of improving life span and quality of life (QoL) [2]. Up to 82% of CKD patients undergoing hemodialysis (HD) experience chronic pain, and 35–70% of patients describe the pain as moderate to severe [1–3]. Chronic pain in CKD patients undergoing HD may be caused by various factors, including musculoskeletal, ischemic, abdominal, chest, and neuropathic pain [2, 3].

Chronic pain in CKD patients has been considered a genuine neurologic disturbance, and it co-occurs generally with many pain syndromes in the context of suffering throughout the lifetime [4, 5]. Studies suggest that long-term chronic pain may result in the development of adaptive neuroplasticity and functional reorganization, with a network shift from affective processing of salience to cognitive modulation of pain [6]. Pain-related regions are parts of the pain neuromatrix (medial prefrontal cortex, posterior cingulate cortex, and insula) and exhibit abnormal functional and structural changes in chronic pain when compared with those in healthy people [6]. These modulatory system regions are associated with chronic pain, cognitive control of emotion, and self-referential processing [6].

Pain is a major cause of depression, disturbed sleep patterns, impaired dialysis adequacy (if unable to endure full sessions), and likeliness of withdrawal from dialysis [3]. Several researchers have also shown that pain negatively impacts many aspects of QoL, including physical function, social function, daily activities, energy, fatigue, and emotional function [7]. Markers of both muscle mass and strength are important predictors of outcomes in this patient population and a decrease in these markers can contribute to risk of physical disability and cognitive decline [8].

In addition to medical therapies, adjunctive non-pharmacologic interventions must be considered whenever applicable for pain in CKD patients undergoing dialysis. Suboptimal pain control is associated with poor QoL, depression, and possibly long-term survival. Nonetheless, adequate medical pain control remains a challenge due to the potential for drug-induced complications and abuse and dependence. Clinicians and the research community should promote pain management in CKD patients undergoing dialysis as a clinical and research priority to improve their QoL and pain-related disability.

Recent studies have shown that novel neuromodulating techniques can have pain-relieving effects in the treatment of chronic pain [9–11]. Studies using anodal transcranial direct current stimulation (tDCS) of motor cortex (M1) or dorsolateral prefrontal cortex (DLPFC) have shown an improvement in pain and depression in several chronic pain syndromes [9]. Anodal tDCS has been shown to increase cortical excitability, promoting pain relief through indirect effects on pain-processing regions in the brain [12]. Promising results have been reported for pain in several diseases such as fibromyalgia, neuropathic pain, chronic post-stroke pain, low back pain, and pelvic pain [9].

tDCS emerges as a simple potential tool to improve QoL and functionality and shed light on the role of brain reorganization in pain function. For CKD patients with chronic pain, tDCS may represent an important cost-efficient, non-pharmacological treatment for a large number of patients with permanent chronic pain, depression, and disability. We currently have no evidence from randomized controlled clinical trials on the use of tDCS to treat pain and depression in CKD patients undergoing HD.

The primary objective of the current study is to evaluate the effects of anodal tDCS over M1 and DLPFC on pain in CKD patients undergoing HD. The secondary outcomes are to assess its impact on depression, anxiety, physical performance, and QoL.

## Methods

### Study design and recruitment

This protocol is presented in accordance with the SPIRIT Initiative 2013 (Additional file 1), an evidence-based guide for the content of trial protocols. This study is a single-center, parallel, randomized, sham-controlled trial in which patients and assessors will be blinded. Patients with CKD undergoing HD will be recruited at Instituto do Rim by invitation after the benefits of tDCS for chronic pain syndromes is explained to them. To help achieve adequate participant enrolment, flyers will be placed at the recruitment site and physicians will be informed of the study to assist with identifying potentially eligible participants.

This study will be conducted at the Federal University of Rio Grande do Norte, Natal, Brazil and registered with the Brazilian Clinical Trials Registry ReBec (www.ensaiosclinicos.gov.br/rg/RBR-7qk2cs/), registration number U1111–1216-0137; June 20, 2018. Data will be stored at Motricity Lab—Federal University of Rio Grande do Norte and will be archived with the principal investigator for 5 years and then destroyed. Possible changes to the protocol will be communicated to the local Ethics Committee, ReBec, and participants. Documents will not be made public without the permission of the participants and resulting publications will not identify the subjects.

All participants will be informed about the trial’s objectives and procedures and that participation is voluntary, as determined by resolution No. 466/12 of the National Health Council. Potentially eligible patients with CKD undergoing HD who are willing to know more about the study will receive a detailed study explanation from the study research coordinator, including objectives, procedures, risks, and benefits of the study and all questions will be answered. Interested patients will be asked to sign an informed consent form (Additional file 2) before entry into the study. Informed consent will be obtained by the research team before study assessments and study procedures are performed and before any private information is recorded. After agreeing to participate in the trial, all participants will sign the informed consent form, which was submitted and approved by the ethics committee of the Faculty of Health Sciences of Trairí—Federal University of Rio Grande do Norte (number 2.678.737). Adult patients assisted in the dialysis center will be recruited.

After the end of the study all patients who received the sham tDCS will be invited to do the active tDCS. Investigators will record the data collected and confidentiality will be guaranteed. Several post-recruitment retention strategies will be used to retain participants in this study and optimize follow-up data collection rates. All participants will be contacted by telephone and email to remind them of their follow-up assessments. Also, participants will receive email or mobile messages informing them about the contribution they are making by remaining in the study.

### Inclusion criteria

Patients will be included if they fulfill the following inclusion criteria: (1) male or female aged 18 to 75 years; (2) undergoing HD with ESRD (CKD 5D^2^) for > 3 months, (4-h session); (3) pain related with a score of more than 4 (range of scores from 1 to 10) in a visual analog scale (VAS) for > 3 months; (4) have physical capacity to do physical evaluation and be capable of consenting to treatment and understanding study explanations and questionnaires; and (5) provide informed consent.

### Exclusion criteria

Patients with any of the following criteria will be excluded from the study: (1) electrical implants in the body; (2) a history of epilepsy or convulsion; (3) clinically contraindicated to receive tDCS, such as having metal embedded in their scalp or brain; (4) psychiatric illness; (5) pregnant women; and (6) signs of severe disease and/or indication of hospitalization, including hemodynamic instability, infection, acute myocardial infarction, and stroke.

### Chronic kidney disease

According to Brkovic et al. [3], end stage renal disease (ESRD) is defined as loss of renal function requiring renal replacement therapy (RRT) with any form of chronic dialysis or renal transplantation, or occasionally conservative management in the elderly or those with significant comorbidities. Acute kidney injury requiring dialysis is not considered ESRD unless renal function fails to recover [3]. In addition, conservative management should be an option in people with ESRD and this should be supported by a comprehensive management program that includes protocols for symptom and pain management, psychological care, spiritual care, and culturally sensitive care for the dying patient and their family [13].

Dialysis patients report significantly more bodily pain, lower vitality, poorer general health, physical, mental, and social dysfunction, and limitations in their ability to work and participate in activities [14]. To measure health status outcomes, Clinical Practice Guideline for Chronic Kidney Disease recommends use of different methods and instruments to measure pain, QoL, health status, and functional status [14].

### Sample size

Sample size was estimated based on statistical considerations for a parallel trial and on previous studies that have paired noninvasive stimulation with chronic pain syndromes [9, 10, 15]. No previous studies have reported results about tDCS using a visual analog scale (VAS) or numeric rating scale (NRS) in CKD. G-Power 3.1.9.2 was used to calculate sample size. VAS is a 0–10-point scale and will be used as the primary outcome. Sample size calculation assumed a significance of 0.05 and power of 0.80. Fregni suggested in previous studies that a mean reduction of 3 points in VAS for the group under active stimulation was expected in contrast to no improvement in the sham group [15]. Sample size was estimated based on the assumption of significance of 0.05, power of 95%, with 0.40 effect size. According to this methodology the sample size would be 36 participants. We decided to add nine more patients to prevent any reduction of power in case of patient dropout. Thus, 45 patients will be recruited and randomized into three groups of 15 patients each.

### Randomization method

Eligible and consenting patients will be randomized using the order of entry into the study and a previous computer-generated randomization list, using random blocks of six patients (for each six patients, two will be randomized to each group) to minimize the risk of unbalanced group sizes. A research assistant external to the study will generate the allocation sequence. Each participant will be equally likely to belong to any one of the groups. Opaque envelopes will be used to conceal allocations. Participants and researchers involved in the assessments and interventions will be blind to group allocation throughout the trial.

### Blinding

Patients and evaluating researchers will be blinded. To ensure patient blinding, the electrodes of the sham group will be placed in the same position as for active groups. The anodal electrode will be placed over the motor cortex and the cathodal over the contralateral supraorbital area, according to placement procedures described below. Usually when the current initiates, patients report itching or a tingling sensation under the electrodes for 2 or 3 minutes. This method of sham intervention is therefore designed to provide an initial period of tingling so that similar sensations are perceived as during active tDCS [11]. For the placebo group a ramp-up of 30 s and a ramp-down of 30 s will be used. The device displays are identical in active and sham settings. To ensure the success of blinding, we will ask participants and outcome assessors at the endpoint to guess whether the treatment was active or sham. Researchers involved in the assessments will be blinded to group allocation throughout the trial.

### Intervention

**Fig. 1:**
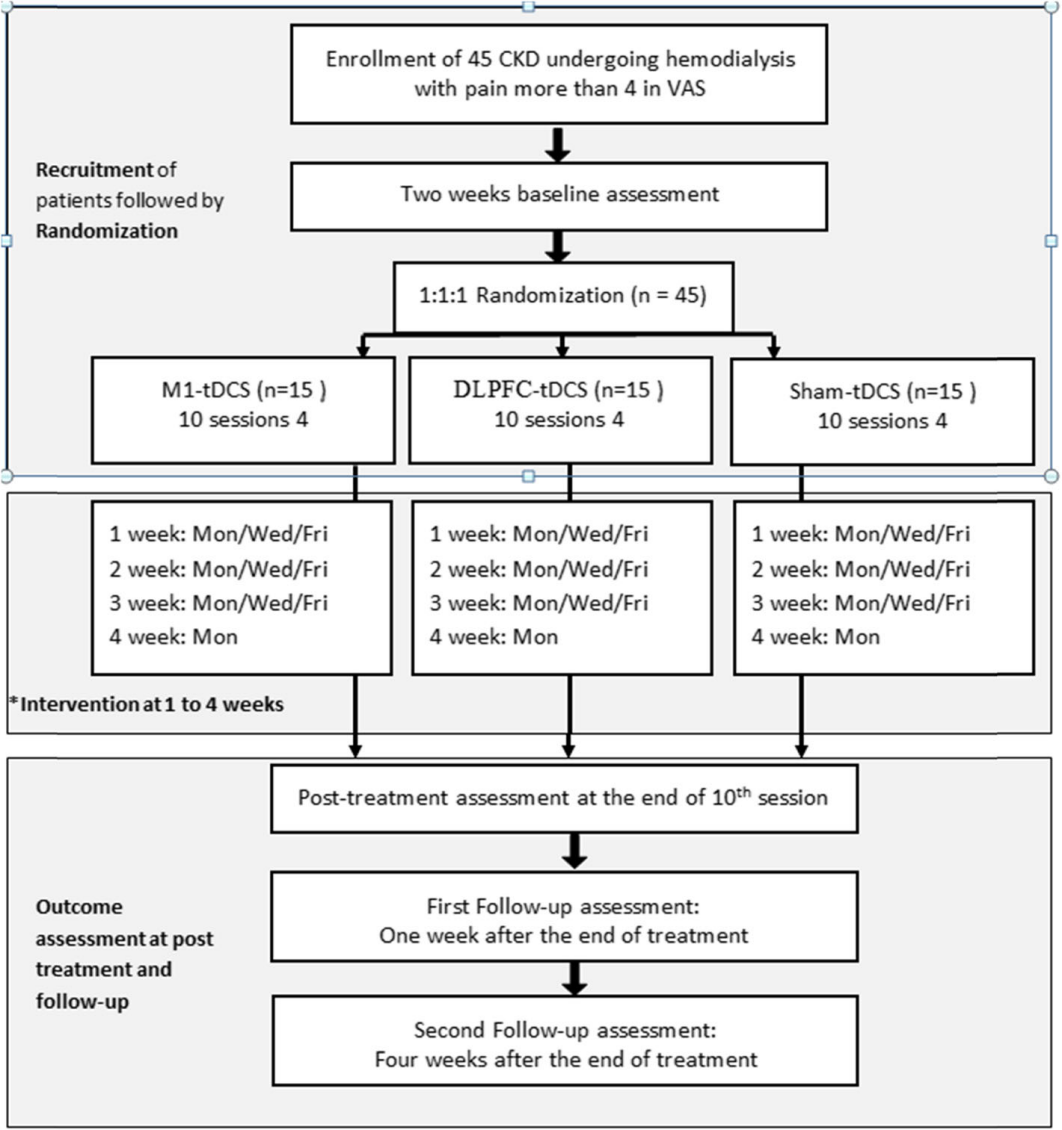
Flowchart summarizing the trial. *According to clinical routine, interventions could be performed on Tuesday/Thursday/Saturday. *VAS* Visual Analogue Scale, *M1* motor cortex, *DLPFC* dorsolateral prefrontal cortex

**Fig. 2:**
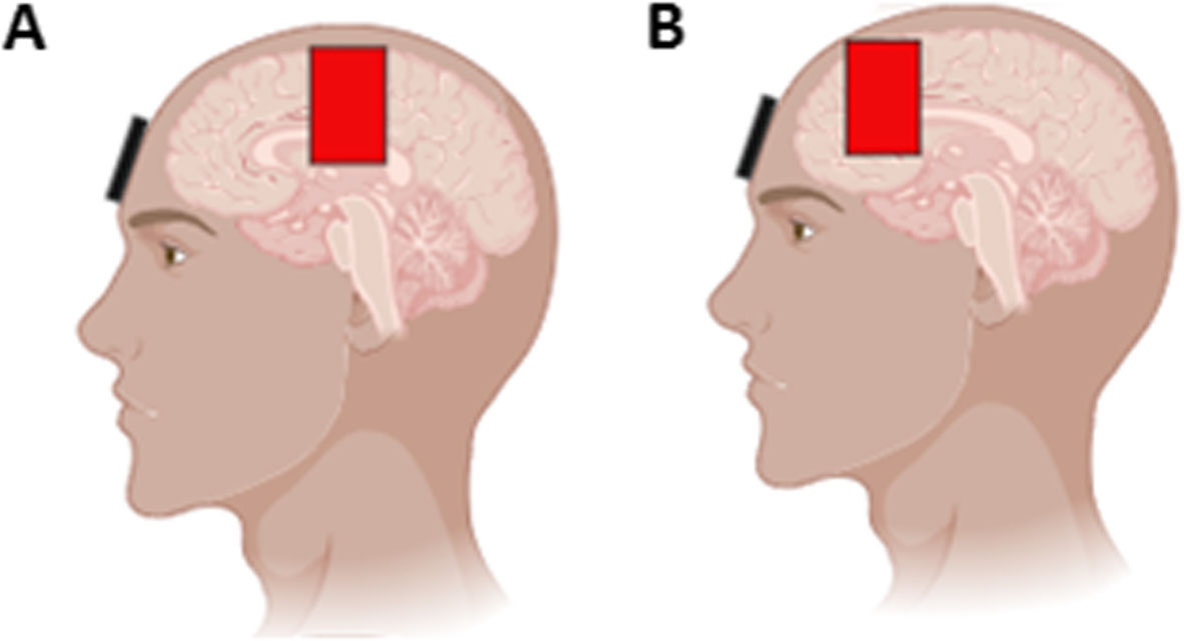
Illustration of electrode replacement. **a** M1 group. The anodal electrode will be placed over the motor cortex and the cathode over the supra-orbital contralateral area (C3/Fp2 montage). **b** DLPFC group. The anodal electrode will be placed over the DLPFC and the cathode over the supra-orbital contralateral area (F3/Fp2 montage). Electrodes will the placed according to 10/20 EEG international system. Electrode size 35 cm^2^ with a current density of 0.057 mA/cm^2^

A total of ten sessions lasting 20 min each will be administered to participants over Monday/Wednesday/Friday or Tuesday/Thursday/Saturday (three sessions per week) by a trained physician at the Instituto do Rim (Fig. 1). The tDCS method will use a monophasic continuous current with an intensity of 2 mA for 20 min (Fig. 2). Each patient will be awake and sit in a comfortable chair with back and arm support during the tDCS intervention. tDCS will be performed before the usual session of dialysis.

During the baseline period, patients will be randomized at a ratio of 1:1:1 to receive sham tDCS (sham group), active tDCS of the primary motor cortex (M1 group), or active tDCS of the left DLPFC (DLPFC group). The M1 was chosen because studies show that stimulation of this area is associated with pain improvement [9, 11, 15, 16]. The left DLPFC was chosen because several studies with neuromodulation have shown that stimulating this area is associated with improvement of depression and also induces an analgesic effect in fibromyalgia [9, 15]. The electrodes will be placed into a 35 cm^2^ square sponge immersed in saline solution (150 mMols of NaCl diluted in water Milli-Q). For stimulation, a gradual current ramp-up and ramp-down with 30-s duration will be used. Electrodes attached to the scalp will be supported by an elastic band. The electrodes (anode and cathode) will be connected to a battery-powered (9 v) stimulator with current verified by a precision digital multimeter (DT832, WeiHua Electronic Co., Ltd, China) with standard error of ±1.5%.

For ethical reasons, no changes will be made to HD routines (days and the place of sessions), clinical care (medicines, imaging, or blood exams), and previous prescription of painkillers or other medication.

### Outcome measures

This study will follow the outcome measures used in studies of multiday repetitive tDCS applied to the M1 or DLPFC to treat chronic pain and depression [17].

**Fig. 3:**
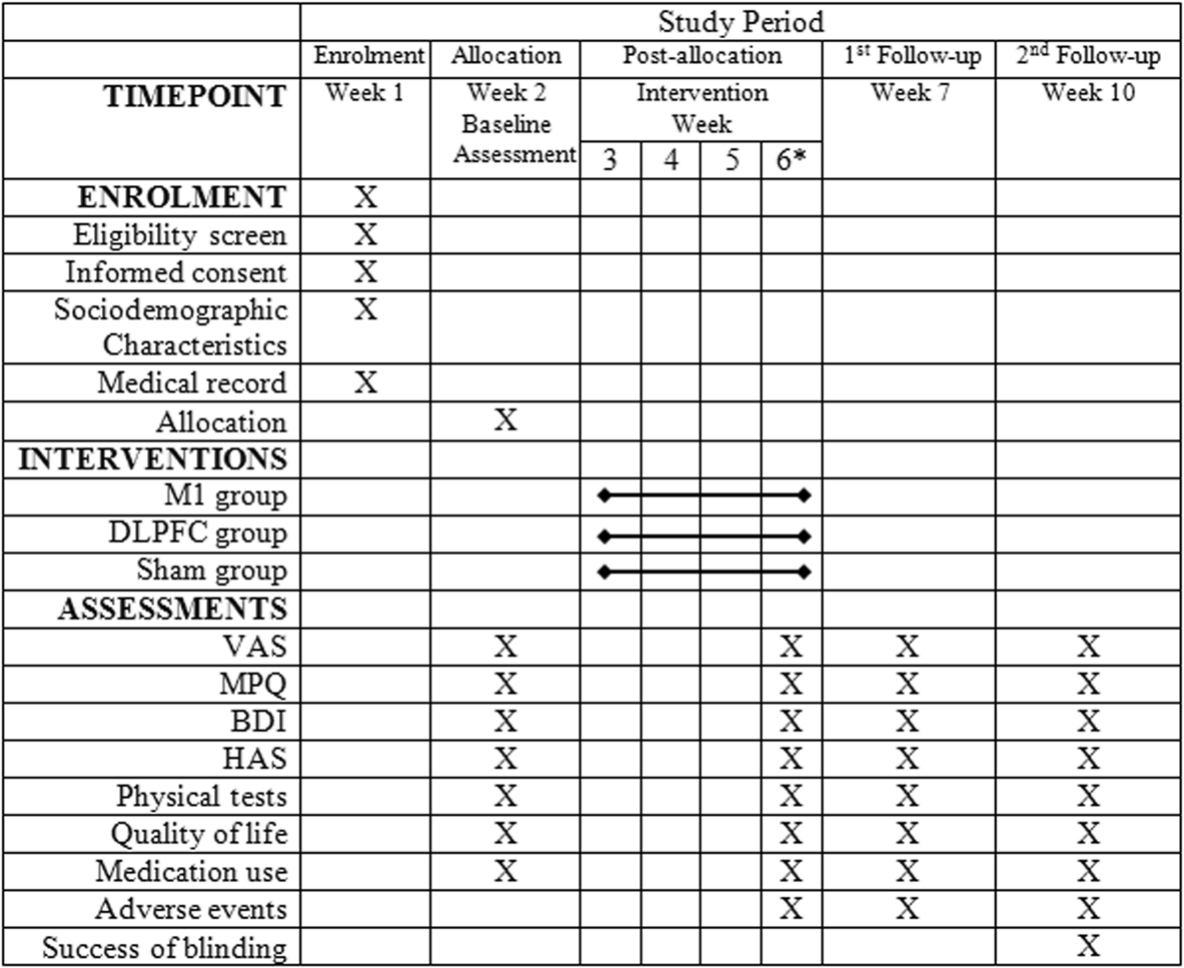
Schedule of enrollment, interventions, and assessments: Recommendations for Interventional Trials (SPIRIT). *VAS* Visual Analogue Scale, *MPQ* McGill Pain Questionnaire, *BDI* Beck Depression Inventory, *HAS* Hamilton Anxiety Scale, *KDQOL-SF* Kidney Disease Quality of Life—Short Form. *Post-intervention evaluation

Data will be collected following assessments at baseline (week 2), immediately after the tenth day of intervention (week 6), and at 7- and 10-week follow-ups (Fig. 3). All evaluations will be performed by an experienced physical therapist blinded to group assignments. Socio-demographic and clinical characteristics will be collected from the participants and will include age, gender, ethnicity, marital status, level of education, income, time of HD, medication profile, and comorbidities.

The primary endpoint of the trial is pain assessed using the VAS pain score and McGill Pain Questionnaire (MPQ). Secondary outcome measures include depression anxiety, physical performance, and quality of life (QoL).

The pain VAS is a unidimensional measure of pain intensity, which has been widely used in diverse adult populations [18]. The pain VAS is a continuous scale consisting of a horizontal line, usually 10 cm (100 mm) in length, anchored by two verbal descriptors, one for each symptom extreme [18]. For pain intensity, the scale is most commonly anchored by “no pain” (score of 0) and “pain as bad as it could be” or “worst imaginable pain” (score of 100 [100-mm scale]). The VAS will be administered and the respondent will be asked to indicate the distance on the 0–100-mm line on the segmented scale that best describes their pain intensity over the last 24 h.

The MPQ is a multidimensional pain questionnaire designed to measure the sensory, affective, and evaluative aspects of pain and pain intensity in adults with chronic pain [18, 19]. The scale contains four subscales evaluating the sensory, affective and evaluative, and miscellaneous aspects of pain, responses to which comprise the Pain Rating Index, and a five-point pain intensity scale [18]. The MPQ is scored by hand by first counting the number of words selected to obtain a number of words chosen score (0–20 words). The Pain Rating Index provides a score ranging from 0 to 78 based on the rank values of the chosen words. The value (score) associated with each descriptor is based on its position or rank order in the word set, such that the first word is given a value of 1, the next a value of 2, and so on [18]. The Pain Rating Index contains 78 pain descriptor items categorized into 20 subclasses, each containing two to six words that fall into four major subscales: sensory (subclasses 1–10), affective (subclasses 11–15), evaluative (subclass 16), and miscellaneous (subclasses 17–20). There is also a one-item pain intensity scale [18]. The MPQ will be assessed by comparing means of total score between groups.

Depression levels will be assessed using the Beck Depression Inventory (BDI), a self-reporting tool of 21 questions relating to cognitive symptoms and attitudes [20]. For each question, patients choose one or more phrases that best describe how they felt in the previous week. The maximum score is 63 points, and high scores indicate severe depression [20]. Beck et al. suggest the following quantification scores for depression: a score of less than 10 indicates minimal or no depression, 10–18 signifies mild to moderate depression, 19–29 moderate to severe depression, and 30–63 severe depression [20]. For analysis, a total value of BDI of each group will be compared.

The severity of anxiety symptoms will be measured using the Hamilton Anxiety Scale (HAS) [21]. The HAS will be administered by an interviewer who asks a series of semi-structured questions related to symptoms of anxiety. The interviewer then will rate the individuals on a five-point scale for each of the 14 items. The values on the scale range from 0 to 4: 0 means that there is no anxiety, 1 indicates mild anxiety, 2 moderate anxiety, 3 severe anxiety, and 4 very severe or grossly disabling anxiety [21]. The total anxiety score ranges from 0 to 56. High levels are indicative of high anxiety.

Strength of the lower limbs will be evaluated by the 30-s chair stand test. A chair 43 cm high, with backrest, without armrest and a stopwatch will be used. Participants will be instructed to keep their arms crossed on their chest and to sit with their backs resting on the backrest. When the signal is given, the participant will stand up and return to the starting position as fast as possible in 30 s. The score corresponds to the number of times that the person is able to perform the complete movement in 30 s.

The arm curl test measures upper body strength and thus may contribute to aerobic fitness and arm muscle endurance. The 30-s arm curl test measures upper-body muscle function and is assessed by the number of arm curl repetitions performed with a 2-kg dumbbell during 30 s.

To assess the flexibility of the lower limbs (posterior thigh muscles), a sit and reach test will be performed. A chair 43 cm high and 50 cm of backrest will be used. During the test, participants will be instructed to sit on the edge of the chair with feet flat on the floor, knees and ankles at 90° flexion; then the dominant or painful leg will be stretched (hip and knee) with the calcaneus supported on the floor and with the ankle flexed at 90°. With overlapping hands and middle fingers on the same level, participants try to get as close as possible to their toes and hold that position for 2 s in three attempts, to get the arithmetic mean. A negative score will be recorded if the middle fingers do not reach the toes, and a score will be positive if the middle fingers were above the toes. Flexibility of the upper limbs will be performed using the scratch flexibility test. Participants will be instructed to pass one hand (dominant or more painful) over the shoulder to assess flexibility of the shoulder in flexion, abduction, and external rotation and to try to reach the other hand to assess extension, adduction, and internal rotation on the center of the back. Three replicates will be made to obtain the arithmetic mean of the results. The measurements will be made using a ruler, and the scores are considered negative if there is any distance between the middle fingers, and positive if the middle fingers overlap.

Timed Up and Go (TUG) test will be used to measure, in seconds, the time taken to stand up from a standard chair, walk 3 m, turn, walk back to the chair, and sit down. A chair with armrests and a seating depth of 44–47 cm is recommended [22]. Those who complete the test in less than 20 s tend to be independently mobile, able to get in and out of a chair without assistance, and climb stairs. The TUG test score correlates well with measures of gait speed, functional ability, and balance. An improvement of 1.4 s on the TUG test (within-patient change score) has been identified as the minimum clinically important difference [22].

The Kidney Disease Quality of Life—Short Form (KDQOL-SF) will be used to assess the QOL. This questionnaire is a specific instrument to evaluate patients on dialysis, applicable to all types of dialysis [23].

A total score for each physical test will be described by means and standard deviations. A total score of variables and all subscales will be compared within and between groups.

### Statistical analysis

Statistical analyses will use SPSS (v.19.0, Chicago, USA) and Graph Pad Prism 5. Clinical and sociodemographic characteristics (marital status, income, education, and ethnicity) will be described by means and standard deviations for continuous numeric parameters, and by frequency tables with 95% confidence intervals for categorical parameters. Chi-squared test will compare the distributions of categorical variables. Kruskal-Wallis test and ANOVA will compare differences in final values between groups.

The Shapiro-Wilk and Levene’s test will assess the normality of the distribution and homogeneity of variance of the data, respectively. Non-parametric tests will be used in case of non-normality distribution. Mauchly’s test of sphericity will be used to validate the correlation of the repeated measures and where the assumption of sphericity is violated, the Greenhouse-Geisser correction will be applied. The effects of stimulation on primary and secondary outcomes will be calculated using a mixed ANOVA model. The dependent variable will be the score of each outcome, and the independent fixed variables will be the time of treatment (baseline, tenth day, first follow-up, and second follow-up), the group of stimulation (active and sham), and the interaction term time vs group. Post-hoc comparisons will be carried out using Bonferroni correction for multiple comparisons.

Friedman test will be used for non-parametric data. Missing data will be treated by intention-to-treat analysis, taking into account the method of the last observation carried forward. Partial η^2^ will be calculated as measures of effect size in the ANOVA results (main effects and interaction effects). Partial η^2^ will be used to calculate the effect size, where η^2^ = 0.01 will be considered small, η^2^ = 0.06 moderate, and η^2^ = 0.14 large effect. Statistical significance will be set at *p* value less than 0.05.

### Adverse event monitoring and adherence

To date, the use of conventional tDCS protocols in human trials (20 min, 2 mA, and ten sessions, in the case of this trial) has not produced any reports of serious adverse effects or irreversible injury [24]. Adverse events will be carefully monitored during all steps of the study by asking patients, after each session of stimulation and during the follow-up period, whether they had experienced any adverse events, and the relationship of these events to treatment with tDCS. The most commonly reported minor adverse events include the sensation of tingling or discomfort under the electrode sites, reported in both active and sham conditions [24, 25]. These events include participant ratings of frequency, severity, importance, and associated distress. To evaluate the adverse effects, we will record any musculoskeletal symptoms such as pain and fatigue, itching, tingling, headache, burning, discomfort, or cardiovascular symptoms such as shortness of breath, chest pain, and abnormal increase in blood pressure. Participants will receive care as appropriate for any harm that arises as a result of study participation.

A doctor will be on duty during tDCS sessions to handle any adverse events, including problems with dialysis. The researchers will communicate any important protocol modifications to relevant parties.

The main strategy to improve adherence of patients is to perform tDCS during HD sessions and explain to participants the relevant contribution to the treatment of chronic pain, including the possibility of improving pain. There will also be flexible hours offered for receiving therapy according to the HD, as well as direct contact by telephone with participants confirming the evaluation dates and reinforcing treatment adherence.

## Discussion

This is the first methodologically designed trial to evaluate the effect of tDCS in ESRD patients undergoing HD. Up to 82% of CKD patients undergoing HD have chronic pain associated with depression, anxiety, poorer QoL, and greater limitations in daily activities [14]. Patients with CKD are prescribed a large number of medications that could promote potentially adverse effects on kidney function or complications due to decreased kidney function. Other non-pharmacological management could improve pain and functionality with no major adverse events.

tDCS is a safe, effective, low-cost, and accessible method for treatment of chronic pain syndromes and it is a potential neuromodulatory technique to relieve pain and depression and increase functionality in ESRD patients undergoing HD.

The protocol described in this paper is a randomized controlled trial designed to guide the development of a larger multicentre study to determine the safety, clinical viability, and patient acceptance of tDCS. The choice of a study design involving three sessions per week offers good conditions to use tDCS in real-world conditions. We assume that the design of the research study may not be accessible to all individuals with ESRD undergoing HD. There will be some clinical and social limitations to include patients in this trial, as it is expected that patients have low educational and income levels, and for these reasons, adherence could be impaired.

If a positive effect is demonstrated in pain, mood states, and functionality, tDCS may represent an important alternative and complementary option for non-invasive brain stimulation for ESRD patients undergoing HD.

Thus, new treatments supporting this strategy are required to improve the care of CKD patients undergoing HD suffering from chronic pain.

## Supplementary information

**Additional file 1.** Standard Protocol Items: Recommendations for Interventional Trials (SPIRIT).

**Additional file 2.** Informed consent form.

## Data Availability

Data will be saved in a public database for possible future research and transparency.
