# Supplementary material for: Transcranial direct current stimulation in management of pain, mood, functionality, and quality of life in patients undergoing hemodialysis: a study protocol for a double-blind controlled randomized trial
Source: Trials. 2019 Dec 30;20:805. doi: 10.1186/s13063-019-3769-6 (PMC6937834; doi:10.1186/s13063-019-3769-6)
Supplement: Supplementary file 2 — Additional file 2. Informed consent form. [file 13063_2019_3769_MOESM2_ESM.docx]

**
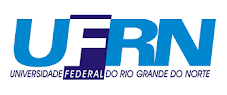
**

FEDERAL UNIVERSITY OF RIO GRANDE DO NORTE

RESEARCH ETHICS COMMITTEES

**Informed Consent Form**

You are being invited to participate to a trial called "Transcranial direct current stimulation (tDCS) and its potentiality in hemodialytic patients", to be carried out at Rio Grande do Norte Federal University, which has Artur Quintiliano Bezerra da Silva (Professor at Federal University of Rio Grande do Norte) as the principal researcher. – artur_bezerra@hotmail.com / telephone +55-84-999915077). This research aims to evaluate the application of a small electric stimulus to the head to improve quality of life and shed light on the role of brain reorganization pain function due to Chronic Kidney Disease (CKD) and may be a promise as a novel cost-efficient non-pharmacological treatment for CKD patients with chronic pain. In this study, we hypothesize that tDCS could improve pain, depression, functionality and quality of life in patients with CKD undergoing hemodialysis. This electrical stimulus is called microcurrent and is so small that it is poorly perceived by the person.

It has been observed that the application of this small electrical stimulus leads to improvement of pain in many diseases, but this treatment was little studied in dialysis patients. Therefore, we would like to evaluate whether there would also be improvement in the clinical condition (pain, symptoms of depression and anxiety and others) in patients undergoing hemodialysis. It is a cost - free and easy - to - apply therapy, where the patient feels little discomfort during therapy and the results were very positive in non - dialytic patients.

If you decide to participate, you should do the following procedures: an initial evaluation through questionnaires assessing anxiety, depression, affection, and about health condition. In addition to these questionnaires, clinical/personal information will be collected: age, marital status, occupation, schooling, family income, race, diseases and medications used. All such information will be collected after your consent and prior to the start of treatment.

After the initial evaluation, the therapy with the microcurrent will be perfomed during 20 minutes (during the dialysis) for 10 days (on dialysis-day), on the scalp through the rubbers, nailed to the head and always accompanied by the research and a nephrologist . This therapy is widely used worldwide and quite safe. During the application of the microcurrent, you may experience itching, heat, a little pain and tingling in the head, but always of slight intensity. These sensations may last up to a few hours after the application of the stimulus.

You have been warned that you can expect some benefits with a safe treatment, already widely used for the treatment of other diseases (long-term pain, Alzheimer's disease, tinnitus in the ear), fast, cost-effective and yet not yet tested in patients undergoing hemodialysis. This therapy may provide in the future, physical benefits as a tolerance to exercise, enhancer of daily activities and quality of life of life. All participants should have access to the results of their process, if interested.

On the other hand, you have received the necessary clarifications about the possible discomforts and risks arising from the study. Positive or negative results will only be obtained after its completion. If you experience these discomforts, you will be given acetaminophen to eliminate o reduce the symptoms. In case of need, such as pain, tingling or itching, you will be referred to the doctor (nephrologist) at the facility. All exams and medications needed to treat any intercurrences will be offered free of charge to the participant.

1/2

During the whole period of the research, you can ask your questions for the researcher responsible (Artur Quintiliano Bezerra da Silva)

You can refuse to participate or withdraw your consent, at any time of the research, without any penalties and without prejudice to your care. You have the right to remove data and materials from the storage location. All results derived from your data will be made available to you by the researcher.

The data that you will provide us will be confidential and will only be divulged in congresses or scientific publications. No data will be published that can identify you. These data will be kept by the researcher responsible for this research in a safe place and for a period of 5 years at Federal University of Rio Grande do Norte.

If you have any expense for your participation in this survey, you will be refunded by research responsible, just contact him on phone contact or email. If you experience any damage from this study, you will be compensated.

After completion of the project, all participants will be reevaluated, one week and one month after the end of treatment. In the same way of the beginning of the research, the questionnaires of evaluation of anxiety, depression, affectivity and health condition will be reapplied.

If you have any damage during or after this study, you are entitled to compensation.

This decision is conditional on compliance with the ethical determinations of the National Health Council - Resolution 466/12

You can call the Research Ethics Committee (REC) of the, telephone +55-84-999915077 or send an e-mail to cepfacisa@gmail.com or cep@facisa.ufrn.br. The REC of Federal University of Rio Grande do Norte is an interdisciplinary and independent collegiate body, constituted under the terms of Resolution 466/2012 of the National Health Council

This study contains questionnaires about the aspect related to your mental, physical and biological health, but you have the right to not answer the questions, if you feel embarrassed.

This document was printed in two ways: one for you and another for the researcher responsible (Rodrigo Pegado de Abreu Freitas)

After being clarified about the objectives, importance, risks, discomforts and benefits that it will bring to me and have been aware of all my rights, i agree to participate in the research "Transcranial direct current stimulation (tDCS) and its potentiality in hemodyalitic patients" and authorize the disclosure of the information I have provided in congresses and / or scientific publications, as long as no data can identify me.

**_____________________________________________**

**Participant**

|  | 2/4  3/4 |
| --- | --- |
|  |  |

**____________ _________________________________**

**Artur Quintiliano Bezerra da Silva**

**Researcher**

**Natal, August 08, 2018.**

2/2

4/4

6/6
